# Change of the age and performance of swimmers across World Championships and Olympic Games finals from 1992 to 2013 – a cross-sectional data analysis

**DOI:** 10.1186/2193-1801-3-652

**Published:** 2014-11-04

**Authors:** Stefan König, Fabio Valeri, Stefanie Wild, Thomas Rosemann, Christoph Alexander Rüst, Beat Knechtle

**Affiliations:** Institute of Primary Care, University of Zurich, Zurich, Switzerland; Gesundheitszentrum St. Gallen, Vadianstrasse 26, 9001 St, Gallen, Switzerland

**Keywords:** Elite swimmers, Age, Performance, Sex difference

## Abstract

The aims of the present study were to investigate the changes in the age and in swimming performance of finalists in World Championships (1994–2013) and Olympic Games (1992–2012) competing in all events/races (stroke and distance). Data of 3,295 performances from 1,615 women and 1,680 men were analysed using correlation analyses and magnitudes of effect sizes. In the World Championships, the age of the finalists increased for all strokes and distances with exception of 200 m backstroke in women, and 400 m freestyle and 200 m breaststroke in men where the age of the finalists decreased. The magnitudes of the effects were small to very large (mean ± SD 2.8 ± 2.7), but extremely large (13.38) for 1,500 m freestyle in women. In the Olympic Games, the age of the finalists increased for all strokes and distances with exception of 800 m freestyle in women and 400 m individual medley in men. The magnitudes of the effects were small to very large (mean ± SD 4.1 ± 7.1), but extremely large for 50 m freestyle in women (10.5) and 200 m butterfly in men (38.0). Swimming performance increased across years in both women and men for all strokes and distances in both the World Championships and the Olympic Games. The magnitudes of the effects were all extremely large in World Championships (mean ± SD 20.1 ± 8.4) and Olympic Games (mean ± SD 52.1 ± 47.6); especially for 100 m and 200 m breaststroke (198) in women in the Olympic Games. To summarize, in the last ~20 years the age of the finalists increased in both the World Championships and the Olympic Games with some minor exceptions (200 m backstroke in women, 400 m freestyle and 200 m breaststroke in men in World Championships and 800 m freestyle in women and 400 m individual medley in men in Olympic Games) and performance of the finalists improved.

## Background

Peaking during the Olympic Games is a major challenge for swimmers, coaches, and scientists (Issurin et al. 2008). Any information that is reliable and valid is of major interest to the athletes and their support (Issurin et al. 2008). Therefore, a large number of investigations were performed to expand the knowledge of peak performance and the age of peak performance in many different sports disciplines such as running (Schulz and Curnow 1988; Berthelot et al. 2012; Lepers and Cattagni 2012), cycling (Schulz and Curnow 1988; Zingg et al. 2013), swimming (Schulz and Curnow 1988; Tanaka and Seals 1997; Rüst et al. 2012, 2014; Buhl et al. 2013b; Kollarz et al. 2013a, [b]; Vaso et al. 2013; Wolfrum et al. 2013; Allen et al. 2014; Zingg et al. 2014a) and triathlon (Stiefel et al. 2013).

As a landmark in the exploration of age and peak athletic performance in elite pool swimmers, Schulz and Curnow (1988) investigated Olympic gold medal winners competing between 1896 and 1980. For freestyle swimming, the age of peak athletic performance was described to be stable at ~18 years for women and at ~21 years for men for almost a century (Schulz and Curnow 1988). In a very recent study, Allen et al. (2014) described the age of individual peak swimming performance in top 16 swimmers competing in the Olympic Games in 2008 or 2012. More studies were done in the field of the age of peak swimming performance, but most of them focused on freestyle (Berthelot et al. 2012; Buhl et al. 2013b; Kollarz et al. 2013b; Wolfrum et al. 2013; Rüst et al. 2014).

However, there was a large range of different athletic collectives examined in different time periods. For example, there were investigations about freestyle Olympic champions from 1896 to 1980 (Schulz and Curnow 1988) as aforementioned, elite freestyle swimmers during 1980–2009 (Berthelot et al. 2012), freestyle top ten US masters in freestyle in the time period of 1993–2002 (Fairbrother 2007a), and elite Swiss freestyle swimmers between 1994 and 2012 (Rüst et al. 2014). These different time periods might have had an influence on the outcome of the results, while the human species developed over time with changes in body dimensions such as body height, body mass and slenderness (Charles and Bejan 2009), and swimming performance increased continuously across the years (Charles and Bejan 2009; Stanula et al. 2012). Therefore, it is of particular importance to be aware of the different time periods in the various studies. Furthermore, it is of great importance to be aware of the methodology of what is analysed exactly. For example, there are a few longitudinal investigations analysing the individual’s peak performance (Pyne et al. 2004; Berthelot et al. 2012; Allen et al. 2014). That approach is different to what is examined in the present cross-sectional study. We intended to investigate the changes in the age of world class swimmers competing in specific competitions. For that reason, we investigated the age of finalists in the World Championships and the Olympic Games.

In terms of the age of peak swimming performance, a few studies reported that there seemed to be a decrease in peak age with increasing race distance in freestyle (Schulz and Curnow 1988; Berthelot et al. 2012; Wolfrum et al. 2013), backstroke (Kollarz et al. 2013a), and individual medley (Buhl et al. 2013b). However, the opposite was found in running where younger athletes were faster in shorter (Schulz and Curnow 1988; Krzysztof and Mero 2013) and older athletes were faster in longer race distances (Trappe 2007; Leyk et al. 2009; Hoffman 2010; Knechtle et al. 2012). Furthermore, a difference in the age of peak swimming performance between the sexes was observed in several cross-sectional studies (Schulz and Curnow 1988; Rüst et al. 2012; Buhl et al. 2013b; Kollarz et al. 2013a; Vaso et al. 2013).

Regarding the change in the age of peak performance in swimming, the age of peak swimming performance seemed to be stable over time (Schulz and Curnow 1988). Schulz and Curnow (1988) described the age of Olympic gold medal winners to be remarkably stable from 1896 to 1980. However, a wide range of different ages in peak freestyle swimming performance was reported in recent studies (Schulz and Curnow 1988; Tanaka and Seals 1997; Fairbrother 2007a, [b]; Berthelot et al. 2012; Buhl et al. 2013a; Kollarz et al. 2013b; Rüst et al. 2014; Allen et al. 2014; see also Tables 1 and 2). In addition, a difference in the age of peak swimming performance between athletes competing at national and international level was observed. For example, Kollarz et al. (2013b) showed in their cross-sectional analysis of elite freestyle and backstroke swimmers competing between 1994 and 2011 that the age of peak swimming performance was more stable across the years for men competing in 50 m freestyle at international than at national level.

**Table 1:** **Studies investigating the age of peak swimming performance in women (yrs)**

	Reference	Freestyle						Breaststroke		Backstroke		Butterfly		Medley	
♀		50 m	100 m	200 m	400 m	800 m	1,500 m	100 m	200 m	100 m	200 m	100 m	200 m	200 m	400 m
National	Buhl et al. 2013b			19.3	18.6									18.2	18.6
	Kollarz et al. 2013b*	19–20	18–21	19–20						18–20	18				
	Rüst et al. 2014*	18.9–20.4	19.1	19.3	18.7	18.5	18.1–25.0								
	Wolfrum et al. 2013	20–21	20–21	20–21				18–19	18–19						
	Zingg et al. 2014c*	19	18	20								18–20	18–20		
International	Allen et al. 2014	26.1	24.2	22.3	22.3	21.9		22.2	23.7	22.6	21.9	23.1	22.0	21.7	21.4
	Buhl et al. 2013b			19.3	20.3									18.2	20.8
	Kollarz et al. 2013b*	20–25	20–24	19–21						21–22	19–21				
	Schulz and Curnow 1988*		18–20		17–18	16									
	Wolfrum et al. 2013	26–27	24–25	22–23				22–23	22–23						

*Studies investigating the change in the age of peak swimming performance.

**Table 2:** **Studies investigating the age of peak swimming performance in men (yrs)**

	Reference	Freestyle						Breaststroke		Backstroke		Butterfly		Medley	
♂		50 m	100 m	200 m	400 m	800 m	1500 m	100 m	200 m	100 m	200 m	100 m	200 m	200 m	400 m
National	Buhl et al. 2013b			21.4	20.3									21.1	20.8
	Kollarz et al. 2013b*	22–23	22–23	21–22						21–22	21				
	Rüst et al. 2014*	23	22.5	21.4	20.3	20.3	20.3								
	Wolfrum et al. 2013	26–27	22–23	22–23				18–19	16–17						
	Zingg et al. 2014c*	22	23	22								23	21		
International	Allen et al. 2014	25.9	25.3	23.6	22.9		22.9	25.2	24.1	24.5	25.2	24.0	24.3	24.8	22.7
	Buhl et al. 2013b			21.4	18.6									21.1	18.6
	Kollarz et al. 2013b*	23–25	23–24	22–24						24	23				
	Schulz and Curnow 1988*		21–22		19–20		19–21								
	Wolfrum et al. 2013	28–29	28–29	22–23				26–27	20–21						

*Studies investigating the change in the age of peak swimming performance.

Concerning the level of the investigated athletes, several studies examined the age of peak swimming performance in swimmers competing at national level and for specific swimming strokes such as freestyle (Tanaka and Seals 1997; Wolfrum et al. 2013; Buhl et al. 2013b; Donato et al. 2003; Rüst et al. 2014), breaststroke (Koch-Ziegenbein et al. 2013; Wolfrum et al. 2013), backstroke (Kollarz et al. 2013a, [b]), butterfly (Zingg et al. 2014b, [c]), and individual medley (Buhl et al. 2013b; Vaso et al. 2013). However, the information about the age of peak swimming performance with its change is missing for swimmers competing at world class level. To date, the study from Schulz and Curnow (1988) was the first and only investigating the change in the age of peak swimming performance in athletes competing at world class level. However, Schulz and Curnow (1988) investigated only freestyle swimming and only Olympic winners. Nevertheless, to the best of our knowledge, no study examined to date the change of the age of peak swimming performance in all strokes and distances with data of athletes competing at world class level in very recent years out of one data set.

Therefore, the aims of the present study were to investigate potential changes in the age of finalists competing in all strokes and all lengths at world class level such as the World Championships and the Olympic Games across recent years. For better comparability, all strokes and distances were included on condition that they were held in both World Championships and Olympic Games from 1994 to 2013 and 1992 to 2012, respectively. Based on Schulz and Curnow’s results (1988), it was hypothesised for all strokes and distances that (1) the age of finalists would remain stable over time for both women and men, and (2) swimming performance would increase across the years for both women and men.

## Materials and methods

### Ethics

All procedures used in the study were approved by the Institutional Review Board of Kanton St. Gallen, Switzerland with a waiver of the requirement for informed consent of the participants given the fact that the study involved the analysis of publicly available data.

### Data sampling

It was intended to collect current data of female and male finalists from recent years. All data for this study were obtained from the world’s swimming ranking (http://www.swimrankings.net). This web page records all swimming race results according to events such as World Championships and Olympic Games. We started data collection from the year 1992 because previous data seemed not complete for all strokes and distances. Full data were available since 1992 for 991 female and 1,008 male performances (1,999 performances in total) in the World Championships and 624 female and 672 male performances (1,296 performances in total) in the Olympic Games. Regarding the World Championships, there was a difference of 17 performances between women and men due to the missing 1,500 m freestyle in women before 2001 and because of one female swimmer who was disqualified in 400 m individual medley at the World Championship in 2001. In the Olympic Games, there was a difference of 48 performances between both sexes due to the missing races in 1,500 m freestyle during the whole period. All races which were held in both the World Championships (1994–2013) and the Olympic Games (1992–2012) were included. Therefore, we excluded 50 m breaststroke, 50 m backstroke, and 50 m butterfly which were only held in the World Championships. The following races were included and analysed: Freestyle (50 m, 100 m, 200 m, 400 m, 800 m, and 1,500 m), breaststroke (100 m, 200 m), backstroke (100 m, 200 m), butterfly (100 m, 200 m), and individual medley (200 m, 400 m). We analysed all listed strokes and distances in both sexes and in both the World Championships and the Olympic Games for changes in the age and swimming performance of all finalists over the years. The following numbers of finals were analysed: In the World Championships, 18 finals were analysed in freestyle for 50 m, 100 m, 200 m, 400 m, and 800 m (nine finals in both women and men for each distance). A total of 16 finals were analysed in 1,500 m freestyle (seven finals in women and nine finals in men). A total of 18 finals were analysed in 100 m and 200 m breaststroke, backstroke, and butterfly (nine finals in both women and men for each stroke and distance). In 200 m and 400 m individual medley 18 finals were analysed (nine finals in both women and men for both distances). In the Olympic Games, 12 finals were analysed in freestyle for 50 m, 100 m, 200 m, 400 m, and 800 m (six finals in both women and men for each distance). In 1,500 m freestyle, six finals were analysed (all of them in men). A total of 12 finals were analysed in 100 m and 200 m breaststroke, backstroke, and butterfly (six finals in both women and men for each stroke and distance). In 200 m and 400 m individual medley 12 finals were analysed (six finals in both women and men for both distances). This leads to a total of 412 finals with eight finalists in each (250 finals in the World Championships and 162 finals in the Olympic Games). The age of the athletes was calculated from the year of birth given at the web page http://www.swimrankings.net.

### Statistical analysis

Each final (*i.e.* set of 8 finalists) was tested for normal distribution and for homogeneity of variances prior to statistical analyses. Normal distribution was tested using a D’Agostino and Pearson omnibus normality test and homogeneity of variances was tested using a Levene’s test. Data in the text and in the tables are given as mean ± standard deviation (SD). Following Hopkins et al. (2009), we calculated the effect size of changes in age and swimming performance since the use of a *p*-value provides no information about the direction or the size of the effect. Since all data were normally distributed, Pearson correlation analysis was used to determine the slope of the change in age and swimming performance, respectively. To calculate effect sizes, we used the equation  where effect sizes were classified as trivial (<0.2), small (0.2–0.6), moderate (0.6–1.2), large (1.2–2.0), very large (2.0–4.0) and extremely large (>4.0) following Hopkins (2006).

## Results

Between 1992 and 2013, data were available for 3,295 performances, including 1,615 female (49%) and 1,680 male (51%) performances. These performances are divided into 1,999 in the World Championships and 1,296 in the Olympic Games.

### Changes in the age of finalists

In the World Championships, the age of the finalists increased for all strokes and distances with the exception of 200 m backstroke in women, 400 m freestyle in men and 200 m breaststroke in men where the age of the finalists decreased (Table 3). The magnitudes of the effects were trivial for 200 m backstroke men, small for 200 m freestyle women, 100 m backstroke women, 100 m and 400 m freestyle men, 200 m breaststroke men, and 400 m medley men, moderate for 50 m and 100 m freestyle women, 200 m backstroke women, 400 m medley women, and 200 m medley men, large for 100 m breaststroke women, 200 m backstroke women, 200 m freestyle men, very large for 400 m and 800 m freestyle women, 100 m and 200 m butterfly women, 50 m and 1500 m freestyle men, 100 m breaststroke men, 100 m backstroke men, 200 m butterfly men, and extremely large for 1500 m freestyle women, 200 m breaststroke women, 200 m medley women, 800 m freestyle men, and 100 m butterfly men.

**Table 3:** **Age (yrs) of the finalists (mean ± SD) at the World Championships 1994–2013 with correlation coefficient and effect size**

Sex	Stroke	Distance	1994	1998	2001	2003	2005	2007	2009	2011	2013	r-value	Effect size
Women	Freestyle	50 m	20.0 ± 4.0	24.3 ± 3.3	25.6 ± 2.8	24.3 ± 5.5	22.8 ± 3.2	25.0 ± 2.8	26.6 ± 8.0	25.4 ± 5.0	22.3 ± 3.9	0.32	0.94
		100 m	20.4 ± 3.5	22.5 ± 2.6	24.3 ± 3.7	24.0 ± 3.5	21.5 ± 2.7	24.1 ± 3.1	21.9 ± 2.5	24.1 ± 2.6	22.5 ± 3.8	0.29	0.81
		200 m	18.9 ± 2.8	22.8 ± 3.6	21.8 ± 4.9	22.8 ± 3.7	21.1 ± 3.4	22.4 ± 3.7	21.8 ± 1.8	21.0 ± 2.4	21.8 ± 2.8	0.23	0.59
		400 m	20.1 ± 2.6	21.5 ± 4.8	20.9 ± 5.1	20.9 ± 3.4	22.0 ± 4.9	21.4 ± 2.7	20.3 ± 5.1	22.4 ± 0.9	23.1 ± 1.9	0.64	3.55
		800 m	19.5 ± 3.4	21.1 ± 2.0	19.0 ± 0.8	20.3 ± 4.6	22.4 ± 4.7	20.8 ± 2.9	22.0 ± 2.9	21.1 ± 2.2	21.9 ± 3.1	0.64	3.55
		1500 m	-	-	18.4 ± 1.0	20.3 ± 4.6	21.8 ± 4.8	22.1 ± 2.5	22.8 ± 3.4	22.4 ± 4.1	22.6 ± 3.8	0.87	13.38
	Breaststroke	100 m	19.1 ± 3.1	22.8 ± 3.6	18.8 ± 3.3	21.0 ± 3.2	22.9 ± 3.2	22.1 ± 2.7	22.1 ± 3.5	22.8 ± 2.0	20.9 ± 4.0	0.47	1.77
		200 m	19.6 ± 2.3	21.8 ± 4.3	18.6 ± 4.0	19.9 ± 2.8	20.3 ± 2.6	22.3 ± 3.0	22.1 ± 1.8	23.0 ± 2.6	22.4 ± 4.0	0.71	4.89
	Backstroke	100 m	20.5 ± 2.0	21.6 ± 3.2	20.8 ± 3.1	21.5 ± 3.7	22.5 ± 2.3	21.0 ± 3.5	20.4 ± 3.0	22.0 ± 3.9	21.1 ± 3.8	0.14	0.32
		200 m	21.1 ± 3.1	23.0 ± 3.5	18.8 ± 2.8	20.0 ± 2.8	19.9 ± 2.6	21.9 ± 3.0	20.3 ± 3.7	19.4 ± 2.3	20.1 ± 2.9	-0.35	1.07
	Butterfly	100 m	19.6 ± 3.0	21.6 ± 3.5	21.3 ± 3.9	23.3 ± 3.7	22.6 ± 4.0	24.4 ± 3.5	22.3 ± 4.5	22.0 ± 2.3	22.8 ± 3.4	0.57	2.65
		200 m	20.0 ± 2.7	21.6 ± 2.7	21.0 ± 4.1	19.5 ± 1.3	20.2 ± 2.5	22.3 ± 3.4	22.1 ± 3.6	21.7 ± 1.7	22.6 ± 1.7	0.65	3.71
	Medley	200 m	19.5 ± 2.3	20.7 ± 3.6	21.0 ± 3.3	21.1 ± 3.4	20.1 ± 3.7	22.0 ± 2.6	21.5 ± 2.5	21.5 ± 2.9	22.5 ± 4.0	0.80	8.0
		400 m	19.8 ± 2.4	19.6 ± 3.0	20.4 ± 3.2	21.3 ± 3.5	19.8 ± 2.6	20.3 ± 2.8	21.8 ± 3.8	19.3 ± 2.7	20.8 ± 2.6	0.28	0.77
Men	Freestyle	50 m	22.6 ± 1.5	23.3 ± 2.6	23.3 ± 3.7	27.8 ± 3.5	25.7 ± 2.6	24.3 ± 3.2	25.0 ± 2.6	24.5 ± 2.6	27.7 ± 4.6	0.58	2.76
		100 m	23.1 ± 2.7	23.6 ± 2.5	23.6 ± 3.1	25.0 ± 4.2	23.7 ± 3.6	26.1 ± 4.1	25.0 ± 2.6	23.7 ± 2.7	23.1 ± 3.0	0.19	0.46
		200 m	21.8 ± 2.9	21.7 ± 2.2	22.1 ± 2.3	23.2 ± 1.7	22.6 ± 3.6	22.5 ± 4.2	21.5 ± 1.8	23.6 ± 3.1	22.6 ± 3.3	0.43	1.50
		400 m	21.2 ± 2.9	21.1 ± 3.8	21.7 ± 2.6	23.5 ± 3.3	23.1 ± 2.4	23.1 ± 3.3	22.7 ± 1.7	23.5 ± 3.0	17.8 ± 5.5	-0.12	0.27
		800 m	21.7 ± 3.1	20.2 ± 1.5	21.6 ± 2.1	21.1 ± 3.4	20.8 ± 1.8	22.8 ± 3.2	23.6 ± 2.0	23.6 ± 2.5	22.5 ± 3.4	0.72	5.14
		1500 m	21.7 ± 3.1	20.2 ± 1.5	22.0 ± 1.8	21.3 ± 3.2	20.5 ± 2.4	23.2 ± 3.1	22.0 ± 2.4	22.8 ± 2.3	22.8 ± 3.4	0.62	3.26
	Breaststroke	100 m	23.0 ± 2.2	24.6 ± 3.9	21.1 ± 1.5	22.8 ± 1.6	24.1 ± 2.0	24.3 ± 2.3	24.2 ± 2.7	25.2 ± 3.1	25.2 ± 3.8	0.64	3.55
		200 m	23.2 ± 3.2	23.1 ± 2.7	22.7 ± 2.8	22.8 ± 2.6	21.7 ± 2.0	22.5 ± 2.4	23.3 ± 2.9	23.1 ± 3.5	22.5 ± 2.0	-0.19	0.46
	Backstroke	100 m	23.7 ± 1.3	22.5 ± 1.8	22.6 ± 3.0	21.1 ± 2.9	22.1 ± 2.0	24.3 ± 2.8	23.2 ± 2.0	24.2 ± 2.6	25.0 ± 3.4	0.54	2.34
		200 m	22.8 ± 1.8	23.5 ± 1.9	21.8 ± 4.0	23.0 ± 3.0	22.8 ± 2.8	24.6 ± 1.9	23.6 ± 3.4	23.2 ± 3.6	22.1 ± 3.4	0.08	0.17
	Butterfly	100 m	22.5 ± 2.6	22.0 ± 2.8	25.1 ± 3.9	23.8 ± 4.7	24.1 ± 3.8	23.0 ± 1.6	23.3 ± 1.9	25.7 ± 3.2	25.8 ± 3.4	0.67	4.06
		200 m	21.0 ± 2.3	21.8 ± 2.9	23.0 ± 4.0	22.8 ± 4.0	20.6 ± 1.2	22.6 ± 1.8	22.3 ± 2.4	23.3 ± 3.4	24.8 ± 3.0	0.66	3.88
	Medley	200 m	22.1 ± 1.8	25.5 ± 3.0	21.8 ± 3.6	22.5 ± 3.4	22.2 ± 2.5	24.5 ± 3.0	24.0 ± 1.7	25.1 ± 3.4	23.2 ± 4.1	0.32	0.94
		400 m	21.3 ± 2.3	23.5 ± 2.0	20.3 ± 2.5	21.7 ± 3.5	21.2 ± 1.6	23.2 ± 2.1	22.3 ± 1.7	22.0 ± 3.6	22.3 ± 2.9	0.23	0.59

In the Olympic Games, similarly to the World Championships, the age of the finalists increased for all strokes and distance with the exception of 800 m freestyle in women and 400 m individual medley in men (Table 4). The magnitudes of the effects were trivial for 800 m freestyle women, small for 200 m backstroke women, 400 m medley women, and 200 m backstroke men, moderate for 100 m butterfly women, 100 m freestyle men, 200 m breaststroke men, and 400 m medley men, large for 100 m and 200 m freestyle women, 200 m medley women, 100 m backstroke men, 100 m butterfly men and 200 m medley men, very large for 400 m freestyle women, 200 m butterfly women, 50 m and 400 m freestyle men, and extremely large for 50 m freestyle women, 100 m and 200 m breaststroke women, 100 m backstroke women, 200 m, 800, and 1500 m freestyle men, 100 m breaststroke men, and 200 m butterfly men.

**Table 4:** **Age (yrs) of the finalists (mean ± SD) at the Olympic Games 1992–2012 with correlation coefficient and effect size**

Sex	Stroke	Distance	1992	1996	2000	2004	2008	2012	r-value	Effect size
Women	Freestyle	50 m	21.3 ± 2.5	23.6 ± 3.6	26.1 ± 3.6	24.3 ± 4.9	25.5 ± 7.2	26.8 ± 5.1	0.84	10.5
		100 m	20.1 ± 3.5	22.7 ± 3.7	25.7 ± 4.0	23.6 ± 4.0	24.1 ± 3.4	22.8 ± 3.5	0.45	1.63
		200 m	18.8 ± 3.8	21.3 ± 3.8	23.6 ± 4.6	20.8 ± 3.9	21.0 ± 2.7	22.3 ± 2.8	0.46	1.70
		400 m	20.6 ± 2.3	21.8 ± 5.0	21.0 ± 4.4	21.0 ± 1.3	21.0 ± 2.4	23.0 ± 2.7	0.56	2.54
		800 m	20.0 ± 1.8	23.8 ± 3.9	19.3 ± 3.2	22.0 ± 4.0	19.5 ± 3.5	21.7 ± 3.4	-0.05	0.10
		1500 m	-	-	-	-	-	-		
	Breaststroke	100 m	20.0 ± 3.9	21.2 ± 4.2	21.5 ± 5.8	22.8 ± 4.7	21.6 ± 3.3	21.8 ± 3.7	0.68	4.25
		200 m	20.2 ± 4.5	19.6 ± 3.7	18.0 ± 2.7	22.1 ± 3.0	21.7 ± 2.9	23.1 ± 2.2	0.71	4.89
	Backstroke	100 m	18.0 ± 2.3	20.0 ± 4.2	21.8 ± 4.5	22.5 ± 3.2	23.2 ± 2.8	21.5 ± 3.9	0.78	7.09
		200 m	20.0 ± 2.4	22.5 ± 3.5	21.2 ± 3.0	21.1 ± 2.4	20.6 ± 4.1	21.7 ± 3.6	0.18	0.43
	Butterfly	100 m	20.3 ± 3.2	20.5 ± 5.0	25.6 ± 4.7	24.2 ± 5.0	21.3 ± 2.4	22.8 ± 2.2	0.34	1.03
		200 m	21.0 ± 4.4	20.2 ± 3.4	21.2 ± 4.5	23.7 ± 4.7	21.7 ± 3.8	22.0 ± 0.7	0.54	2.34
	Medley	200 m	20.0 ± 1.8	22.7 ± 2.7	23.0 ± 3.1	21.0 ± 4.4	21.7 ± 2.4	23.0 ± 3.7	0.43	1.50
		400 m	19.2 ± 1.4	21.0 ± 3.4	20.2 ± 3.2	20.1 ± 2.7	19.2 ± 3.3	20.8 ± 2.7	0.19	0.46
Men	Freestyle	50 m	24.8 ± 2.7	22.1 ± 1.9	25.0 ± 3.6	26.9 ± 3.6	25.0 ± 2.8	27.1 ± 3.6	0.65	3.71
		100 m	24.1 ± 2.7	22.5 ± 2.8	25.3 ± 2.4	24.4 ± 2.8	25.9 ± 4.0	23.8 ± 2.7	0.35	1.07
		200 m	21.5 ± 3.3	22.0 ± 3.3	22.6 ± 3.2	23.1 ± 2.5	22.2 ± 2.1	23.0 ± 2.8	0.75	6.00
		400 m	22.1 ± 3.9	21.5 ± 3.8	19.8 ± 4.8	22.1 ± 2.4	22.9 ± 2.8	23.6 ± 4.3	0.58	2.76
		800 m	22.1 ± 3.0	21.2 ± 2.4	21.5 ± 2.6	22.7 ± 3.2	22.5 ± 3.3	23.1 ± 3.1	0.73	5.40
		1500 m	22.1 ± 2.9	21.3 ± 2.5	21.5 ± 2.7	22.8 ± 3.1	22.5 ± 3.2	23.2 ± 3.0	0.73	5.41
	Breaststroke	100 m	22.5 ± 3.5	23.8 ± 2.4	20.6 ± 2.0	23.1 ± 1.4	26.2 ± 1.7	26.3 ± 3.3	0.70	4.66
		200 m	22.3 ± 2.4	24.6 ± 3.0	23.0 ± 2.5	20.5 ± 2.6	23.6 ± 2.8	25.0 ± 2.9	0.25	0.66
	Backstroke	100 m	23.2 ± 2.6	24.3 ± 2.2	22.8 ± 1.8	22.6 ± 3.9	23.5 ± 1.2	25.7 ± 2.7	0.44	1.57
		200 m	21.6 ± 1.0	23.7 ± 2.0	23.5 ± 4.7	22.7 ± 2.0	24.7 ± 3.3	22.1 ± 3.1	0.22	0.56
	Butterfly	100 m	24.0 ± 2.6	22.8 ± 2.6	21.0 ± 5.1	24.7 ± 3.9	24.1 ± 2.2	25.1 ± 2.9	0.47	1.77
		200 m	21.2 ± 3.2	21.6 ± 1.8	23.1 ± 4.1	22.7 ± 4.4	23.6 ± 1.7	24.1 ± 3.4	0.95	38.0
	Medley	200 m	21.6 ± 3.2	22.7 ± 1.0	25.8 ± 2.0	20.6 ± 2.0	23.5 ± 1.1	25.5 ± 3.7	0.42	1.44
		400 m	23.3 ± 1.1	24.3 ± 2.4	21.2 ± 3.0	20.8 ± 1.9	23.4 ± 2.3	22.2 ± 4.4	-0.35	1.07

### Changes in swimming performance across the years

Swimming performance increased across the years in both women and men for all strokes and distances in the World Championships (Table 5) and the Olympic Games (Table 6). The magnitudes of the effects were all extremely large; especially for 100 m and 200 m breaststroke (198) in women in the Olympic Games.

**Table 5:** **Race times (s) of the finalists (mean ± SD) at the World Championships 1994–2013 with correlation coefficient and effect size**

Sex	Stroke	Distance	1994	1998	2001	2003	2005	2007	2009	2011	2013	r-value	Effect size
Women	Freestyle	50 m	25.32 ± 0.40	25.60 ± 0.31	25.09 ± 0.37	25.13 ± 0.32	25.05 ± 0.30	24.84 ± 0.24	24.07 ± 0.23	24.56 ± 0.24	24.48 ± 0.29	-0.86	12.28
		100 m	55.12 ± 0.73	55.57 ± 0.64	55.12 ± 0.41	54.76 ± 0.23	54.80 ± 0.30	54.05 ± 0.47	53.16 ± 0.56	53.77 ± 0.29	53.53 ± 0.69	-0.90	18.00
		200 m	119.26 ± 1.83	120.07 ± 0.70	119.14 ± 0.41	118.99 ± 0.53	119.18 ± 0.43	117.78 ± 1.97	115.80 ± 1.36	116.70 ± 0.88	116.43 ± 1.06	-0.88	14.66
		400 m	251.77 ± 1.67	249.88 ± 2.22	250.78 ± 2.47	249.59 ± 2.72	247.93 ± 1.22	245.69 ± 1.70	242.73 ± 2.64	245.41 ± 2.48	245.60 ± 2.17	-0.88	14.66
		800 m	514.70 ± 5.02	515.64 ± 6.42	513.93 ± 6.36	512.19 ± 8.04	512.05 ± 5.22	507.35 ± 6.06	501.25 ± 5.28	505.03 ± 5.51	503.29 ± 7.73	-0.92	23.00
		1500 m	-	-	979.34 ± 14.31	974.10 ± 8.43	974.07 ± 8.79	971.97 ± 17.20	959.95 ± 11.19	962.75 ± 7.63	954.73 ± 12.84	-0.95	38.00
	Breaststroke	100 m	70.04 ± 1.15	68.76 ± 0.26	68.65 ± 0.84	68.09 ± 0.83	67.72 ± 0.93	67.62 ± 1.16	66.05 ± 0.69	66.79 ± 0.91	66.11 ± 1.06	-0.95	38.00
		200 m	149.00 ± 1.41	147.42 ± 1.71	145.91 ± 1.02	146.28 ± 2.02	147.10 ± 2.43	146.56 ± 2.20	142.58 ± 0.77	144.80 ± 1.97	142.57 ± 1.94	-0.85	11.33
	Backstroke	100 m	61.64 ± 0.77	61.96 ± 0.60	61.60 ± 0.76	61.16 ± 0.39	61.08 ± 0.49	60.71 ± 0.98	59.05 ± 0.62	59.43 ± 0.41	59.50 ± 0.61	-0.91	20.22
		200 m	131.94 ± 2.88	132.81 ± 1.21	131.42 ± 1.23	131.13 ± 1.77	131.16 ± 1.54	129.57 ± 1.64	126.98 ± 1.53	127.68 ± 1.39	128.34 ± 2.15	-0.90	18.00
	Butterfly	100 m	60.42 ± 0.84	59.32 ± 0.65	59.25 ± 0.58	58.81 ± 0.59	58.56 ± 0.82	58.13 ± 0.79	56.83 ± 0.52	57.41 ± 0.42	57.56 ± 0.64	-0.93	26.57
		200 m	131.74 ± 3.24	130.81 ± 2.24	129.06 ± 1.57	129.28 ± 1.79	129.12 ± 2.19	128.45 ± 2.41	124.75 ± 0.98	126.08 ± 0.42	126.30 ± 1.33	-0.91	20.22
	Medley	200 m	135.63 ± 1.61	134.84 ± 1.91	133.62 ± 1.27	133.93 ± 1.77	133.65 ± 1.97	133.19 ± 2.10	128.75 ± 1.67	130.73 ± 2.40	130.28 ± 1.30	-0.88	14.66
		400 m	284.17 ± 3.48	282.01 ± 4.19	281.91 ± 3.91	282.79 ± 4.04	281.68 ± 2.77	281.83 ± 4.32	274.20 ± 2.82	275.36 ± 2.22	274.04 ± 3.28	-0.87	13.38
Men	Freestyle	50 m	22.62 ± 0.25	22.54 ± 0.19	22.28 ± 0.14	22.29 ± 0.16	22.12 ± 0.26	22.05 ± 0.13	21.36 ± 0.16	21.92 ± 0.17	21.62 ± 0.19	-0.89	16.18
		100 m	49.79 ± 0.39	49.62 ± 0.44	48.96 ± 0.44	49.02 ± 0.42	48.72 ± 0.46	48.56 ± 0.14	47.40 ± 0.38	48.05 ± 0.21	48.05 ± 0.30	-0.90	18.0
		200 m	108.79 ± 0.77	109.39 ± 1.12	107.55 ± 1.88	107.87 ± 1.52	107.03 ± 1.23	106.99 ± 1.53	104.73 ± 1.53	105.63 ± 1.18	105.86 ± 0.90	-0.89	16.18
		400 m	229.96 ± 3.11	229.04 ± 2.21	227.20 ± 4.53	227.55 ± 2.87	226.80 ± 2.37	226.43 ± 1.61	223.75 ± 2.73	224.70 ± 1.65	226.24 ± 2.57	-0.85	11.33
		800 m	483.03 ± 7.51	482.94 ± 5.02	472.20 ± 8.18	475.49 ± 7.21	470.80 ± 7.70	471.36 ± 3.55	463.10 ± 6.48	465.86 ± 4.96	466.99 ± 4.42	-0.89	16.18
		1500 m	911.14 ± 13.18	909.55 ± 11.26	904.14 ± 13.64	904.83 ± 10.17	897.97 ± 12.25	895.01 ± 7.19	893.29 ± 13.28	894.98 ± 16.20	892.54 ± 9.69	-0.95	38.00
	Breaststroke	100 m	61.84 ± 0.41	62.16 ± 0.54	61.06 ± 0.68	60.79 ± 0.61	60.46 ± 0.74	60.73 ± 0.63	59.06 ± 0.33	59.84 ± 0.59	59.61 ± 0.48	-0.90	18.00
		200 m	134.25 ± 0.99	134.36 ± 0.93	131.65 ± 0.89	131.89 ± 1.39	131.91 ± 0.99	131.31 ± 0.73	128.44 ± 0.95	129.94 ± 1.21	129.02 ± 0.88	-0.91	20.22
	Backstroke	100 m	55.73 ± 0.30	55.41 ± 0.38	55.14 ± 0.52	54.39 ± 0.57	54.40 ± 0.46	53.98 ± 0.70	52.79 ± 0.36	53.07 ± 0.25	53.47 ± 0.43	-0.93	26.57
		200 m	119.42 ± 1.05	119.91 ± 0.78	118.90 ± 1.01	117.85 ± 0.91	117.49 ± 1.50	117.12 ± 1.95	114.43 ± 1.59	116.01 ± 1.84	115.52 ± 1.49	-0.91	20.22
	Butterfly	100 m	53.96 ± 0.38	53.12 ± 0.48	52.62 ± 0.39	51.94 ± 0.71	52.32 ± 0.95	51.93 ± 0.75	50.75 ± 0.67	51.61 ± 0.55	51.49 ± 0.19	-0.87	13.38
		200 m	118.62 ± 1.21	118.23 ± 1.37	116.20 ± 1.28	116.54 ± 1.50	116.66 ± 0.94	115.51 ± 1.69	114.09 ± 1.34	114.81 ± 0.77	115.54 ± 0.72	-0.86	12.28
	Medley	200 m	122.20 ± 2.06	122.63 ± 1.54	121.26 ± 0.81	119.98 ± 1.79	119.63 ± 1.96	118.33 ± 2.03	116.52 ± 1.76	117.44 ± 2.18	117.28 ± 1.49	-0.95	38.00
		400 m	257.88 ± 3.82	258.53 ± 2.90	257.96 ± 3.10	255.95 ± 4.22	254.58 ± 3.33	252.77 ± 3.99	250.06 ± 2.83	253.74 ± 3.77	251.67 ± 3.02	-0.88	14.66

**Table 6:** **Race times (s) of the finalists (mean ± SD) at the Olympic Games with correlation coefficient and effect size**

Sex	Stroke	Distance	1992	1996	2000	2004	2008	2012	r-value	Effect size
Women	Freestyle	50 m	25.36 ± 0.33	25.36 ± 0.37	25.01 ± 0.47	24.96 ± 0.18	24.35 ± 0.28	24.44 ± 0.20	-0.94	31.33
		100 m	55.46 ± 0.67	55.37 ± 0.59	54.75 ± 0.62	54.57 ± 0.46	53.80 ± 0.49	53.54 ± 0.30	-0.98	98.01
		200 m	119.94 ± 1.43	119.95 ± 1.26	118.92 ± 0.48	118.69 ± 0.43	116.35 ± 1.34	116.26 ± 1.35	-0.94	31.33
		400 m	251.54 ± 3.05	250.00 ± 2.16	249.46 ± 2.55	248.05 ± 2.56	244.89 ± 2.66	244.14 ± 1.94	-0.98	98.00
		800 m	514.78 ± 5.40	514.89 ± 5.48	507.82 ± 5.74	509.05 ± 4.39	504.41 ± 5.69	502.84 ± 500	-0.95	38.00
		1500 m	-	-	-	-	-	-		
	Breaststroke	100 m	69.56 ± 1.05	69.02 ± 0.72	68.16 ± 0.92	67.54 ± 0.62	67.30 ± 0.99	66.60 ± 0.73	-0.99	198.0
		200 m	148.97 ± 2.05	147.73 ± 1.90	145.64 ± 1.04	145.48 ± 1.25	143.30 ± 1.63	142.37 ± 2.05	-0.99	198.0
	Backstroke	100 m	61.81 ± 0.77	62.20 ± 0.58	61.12 ± 0.58	61.07 ± 0.48	59.53 ± 0.43	59.13 ± 0.63	-0.93	26.57
		200 m	131.23 ± 2.21	132.69 ± 2.21	131.70 ± 1.98	130.69 ± 1.28	127.77 ± 1.54	127.21 ± 1.75	-0.86	12.28
	Butterfly	100 m	59.60 ± 0.92	59.89 ± 0.64	58.47 ± 0.91	58.54 ± 0.72	57.70 ± 0.60	57.10 ± 0.53	-0.95	38.00
		200 m	130.49 ± 1.76	130.46 ± 1.38	128.18 ± 1.60	128.58 ± 1.72	126.58 ± 1.43	126.15 ± 1.23	-0.95	38.00
	Medley	200 m	135.22 ± 2.56	135.59 ± 1.19	133.50 ± 1.48	133.24 ± 1.35	130.85 ± 1.72	130.08 ± 2.10	-0.96	48.00
		400 m	283.68 ± 6.18	283.47 ± 2.20	280.88 ± 4.73	280.99 ± 6.27	275.15 ± 4.84	273.35 ± 2.49	-0.94	31.33
Men	Freestyle	50 m	22.38 ± 0.26	22.46 ± 0.23	22.18 ± 0.19	22.11 ± 0.15	21.56 ± 0.14	21.68 ± 0.20	-0.92	23.00
		100 m	49.58 ± 0.35	49.30 ± 0.42	48.94 ± 0.39	48.80 ± 0.46	47.77 ± 0.39	47.87 ± 0.29	-0.96	48.00
		200 m	108.19 ± 1.22	108.39 ± 0.49	107.43 ± 1.50	106.49 ± 1.38	105.81 ± 1.48	105.59 ± 1.44	-0.97	64.66
		400 m	227.82 ± 1.96	230.56 ± 2.03	226.21 ± 2.76	225.91 ± 2.26	223.71 ± 1.97	225.52 ± 3.12	-0.74	5.69
		800 m	481.20 ± 7.43	483.08 ± 5.20	477.99 ± 4.09	477.50 ± 4.23	473.01 ± 2.42	473.45 ± 5.12	-0.92	23.00
		1500 m	908.89 ± 15.84	910.29 ± 9.17	901.67 ± 10.15	898.04 ± 12.61	888.60 ± 8.47	887.69 ± 9.75	-0.96	48.00
	Breaststroke	100 m	61.92 ± 0.29	61.46 ± 0.53	61.25 ± 0.49	61.14 ± 0.75	59.63 ± 0.46	59.61 ± 0.711	-0.94	31.33
		200 m	132.83 ± 1.76	134.31 ± 1.27	132.84 ± 0.91	131.22 ± 0.85	129.44 ± 1.16	128.54 ± 0.84	-0.91	20.22
	Backstroke	100 m	55.12 ± 0.82	55.18 ± 0.51	54.85 ± 0.65	54.52 ± 0.35	53.27 ± 0.40	53.16 ± 0.50	-0.93	26.57
		200 m	119.59 ± 0.73	120.87 ± 3.07	118.42 ± 1.02	117.98 ± 1.52	115.54 ± 1.08	115.62 ± 1.80	-0.91	20.22
	Butterfly	100 m	53.82 ± 0.40	53.07 ± 0.44	52.53 ± 0.41	51.98 ± 0.57	51.23 ± 0.46	51.68 ± 0.28	-0.94	31.33
		200 m	118.56 ± 1.06	117.97 ± 0.80	116.68 ± 1.05	115.89 ± 1.17	113.85 ± 1.12	114.24 ± 1.01	-0.97	64.66
	Medley	200 m	121.90 ± 1.19	121.86 ± 1.60	121.12 ± 1.22	119.71 ± 1.62	117.88 ± 2.06	117.02 ± 1.83	-0.97	64.66
		400 m	258.46 ± 3.44	257.59 ± 2.29	256.73 ± 3.16	254.70 ± 4.37	250.54 ± 4.02	250.79 ± 3.25	-0.96	48.00

## Discussion

The present study investigated potential changes in the age of finalists and in swimming performance in all strokes and distances in indoor pool swimming for both women and men competing at world class level. The main findings were, firstly, the age of the finalists increased across years for both women and men in both the World Championships and the Olympic Games with some minor exceptions and, secondly, swimming performance improved for all distances and strokes.

### Change in the age of finalists over time

The age of the finalists increased for all strokes and distances with the exception of 200 m backstroke in women, 400 m freestyle in men and 200 m breaststroke in men in the World Championships and 800 m freestyle in women and 400 m individual medley in men in the Olympic Games where the age of the finalists decreased. This finding disagrees with our hypothesis on the basis of a few studies in the field investigating changes in the age of peak performance across years in swimmers competing at different levels (Schulz and Curnow 1988; Rüst et al. 2012; Kollarz et al. 2013b; Zingg et al. 2014c).

The trend of an increase in the age of peak performance across years is in line with observations in different sports disciplines such as swimming (Knechtle et al. 2014), running (Lepers and Cattagni 2012) and triathlon (Meili et al. 2013; Gallmann et al. 2014) where the athletes get older with continuous improvement in performance. For example, in long-distance triathletes competing in the ‘Ironman Hawaii’ the age of peak triathlon performance increased from 26 ± 5 to 35 ± 5 years for women and from 27 ± 2 to 34 ± 3 years for men between 1983 and 2012 with a decrease of race times (Gallmann et al. 2014). Similarly, in ultra-distance triathletes competing in ‘Ultraman Hawaii’, the annual top three women and men improved their performance during the 1983–2012 period although the age of the annual top three women and men increased (Meili et al. 2013). The age of the annual top three finishers increased from 33 ± 6 to 48 ± 3 years for men and from 29 ± 7 to 49 ± 2 years for women (Meili et al. 2013). In ultra-swimmers competing between 1983 and 2013 in the 46 km ‘Manhattan Island Marathon Swim’, the age of the annual three fastest swimmer increased from 28 ± 4 to 38 ± 6 years in women and from 23 ± 4 to 42 ± 8 years in men although race times remained stable across years (Knechtle et al. 2014).

Although the age of the fastest finalists increased in most events/races (stroke and distance), the age of the finalists decreased in 200 m backstroke women, 200 m breaststroke men and 400 m freestyle men in the World Championships and in 400 m individual medley men and 800 m freestyle women. A common trend for these events and races is that they are all 200 m in length and longer. In a number of studies it was reported for a few races that there was a change in the age of peak swimming performance across the years (Rüst et al. 2012; Kollarz et al. 2013b; Zingg et al. 2014c). Generally, the age of the finalists increased with the exception for swimmers competing at national level that the age of peak swimming performance decreased for women and increased for men in 50 m freestyle from 2006 to 2010 (Rüst et al. 2012). Furthermore, changes in the age of peak performance were mainly reported for shorter distances such as the 50 m distance. For backstroke, Kollarz et al. (2013b) showed that the age of peak swimming performance increased in 50 m backstroke for women from ~16 to ~22 years and in 50 m freestyle for men from ~22 to ~23 years in swimmers competing at national level from 1994 to 2011. Furthermore, Zingg et al. (2014c) reported that the age of peak swimming performance for athletes competing at national level between 1994 and 2011 increased for women in 50 m butterfly from ~19 to ~21 years, in 100 m and 200 m butterfly from ~18 to ~20 years, while it was stable in all race lengths of butterfly for men. These studies confirm our observation that there is a trend of an increase in the age of peak swimming performance over time.

However, as a basis for our hypothesis, several authors reported that the age of peak swimming performance remained stable over time (Schulz and Curnow 1988; Berthelot et al. 2012; Buhl et al. 2013b; Kollarz et al. 2013b; Rüst et al. 2014). For example for swimmers competing at world class level, Schulz and Curnow (1988) described the age of Olympic gold medal winners from 1896 to 1980 to be remarkably stable for women and men. However, Schulz and Curnow (1988) did not investigate 50 m for both women and men, and 800 m in men. Furthermore, Buhl et al. (2013a) showed that the age of peak swimming performance remained unchanged in individual medley in elite Swiss swimmers competing from 1994 to 2011. However, Kollarz et al. (2013b) observed in their comparison of elite freestyle and backstroke swimmers that the age of peak swimming performance was more stable at international than at national level between 1994 and 2011. They reported that the age of peak freestyle and backstroke swimming performance increased in 50 m for women and men at national level. On the other hand, there were no changes observed in swimmers at international level in all examined distances of 50 m, 100 m, and 200 m in freestyle and backstroke. Thus, we have to be aware that the studies mentioned above might therefore be affected by this difference with their swimmers’ collective at national level.

### Increase of peak swimming performance across the years

Swimming performance improved for all distances and strokes in both the World Championships and the Olympic Games. This finding fully agrees with our hypothesis based on studies about the progression of swimming performance in different strokes and distances (Schulz and Curnow 1988; Buhl et al. 2013a; Koch-Ziegenbein et al. 2013; Kollarz et al. 2013b; Zingg et al. 2014c). Thus, a further increase in swimming performance would be expected. However, we might not have examined a time period that is large enough to assess progression in swimming performance with validity. Some investigations revealed that swimming performance would reach its limit (Nevill et al. 2007; Berthelot et al. 2012; Stanula et al. 2012). Nevill et al. (2007) notified that a non-linear, flattened S-shaped logistic curve would better describe the world record performances in front-crawl swimming from 1957 to 2007. With regard to the examined time period it is likely that the results of the present study and all the listed investigations are lacking validity. Berthelot et al. (2012) described that swimming performances might soon reach their limits. These authors reported that swimming speed still progressed until recent days of the study (Berthelot et al. 2010). Stanula et al. (2012) showed a limit in peak swimming performance in 50 m to 800 m freestyle, whereas Buhl et al. (2013a) reported that swimming performance remained unchanged in 200 m freestyle in women competing at international level. Nevertheless, swimming performance increased linearly up to 5.3% in 200 m and 400 m freestyle and in 400 m individual medley at national (*i.e.* Switzerland) and international level in the other races than mentioned above (*i.e.* World Championship finals) during the time period of 1994 to 2011 (Buhl et al. 2013a). For breaststroke, Koch-Ziegenbein et al. (2013) described an increase in swimming performance of 1.2% to 5.2% in all strokes and distances except for 50 m backstroke in women competing at international level. For butterfly, Zingg et al. (2014c) showed that swimming performance increased over all distances in both women and men. The trend in progression of peak swimming performance remains a controversial question and needs further investigation.

New World and Olympic swimming records were set continuously with a peak rate of new records at the World Championship in 2009 as a major result of the newly introduced swimsuits in those days (O’Connor and Vozenilek 2011). The best swimming performances, according to swimming speed, so far were held by Britta Steffen and Cesar Cielo with 23.73 sec (2.11 m ·s^-1^) and 21.08 sec (2.37 m ·s^-1^), respectively, both at the World Championship in 2009 (http://www.fina.org). There are several studies investigating swimsuits, modern wetsuits and fast-skin body suit (Toussaint et al. 1989; Toussaint et al. 2002; Tomikawa and Nomura 2009; Chatard and Wilson 2008; Tiozzo et al. 2009). The effect of these specialized swimsuits were described as a reduction of drag force by 14% at an average swimming velocity of 1.25 m ·s^-1^ (Toussaint et al. 1989), better results in swimming performances when wearing a wetsuit compared to a conventional swimsuit (Tomikawa and Nomura 2009), and improvements in swimming performance while wearing fast-skin suit versus normal swimsuit (Toussaint et al. 2002) and full-body suit or waist-to-ankle suit compared to a normal swimsuit (Chatard and Wilson 2008). In 2009, there was no doubt anymore about the benefit of wearing a bodysuit (Tiozzo et al. 2009). The way was paved for further steps in the sport of swimming. Thus, FINA banned those modern swimsuits from January 1st, 2010, onward (http://www.fina.org). The equipment is an important factor in reaching peak swimming performance. However, there are many different factors according to the development of higher swimming performance, which are relevant in this field.

Different parameters play a role in the development of swimming performance where the main factors are anthropometric characteristics (Siders et al. 1993; Jagomägi and Jürimäe 2005; Zampagni et al. 2008; Charles and Bejan 2009; Lätt et al. 2010), biomechanical (Jagomägi and Jürimäe 2005; Zampagni et al. 2008) and physiological parameters (Tanaka and Seals 2003; Zamparo 2006; Jürimäe et al. 2007). Over the years, the human species developed with a change of anthropometry such as body height, body weight, and slenderness (Siders et al. 1993; Jagomägi and Jürimäe 2005; Zampagni et al. 2008; Charles and Bejan 2009) that might affect swimming performance. Charles and Bejan (2009) reported a growth of champion swimmers by 11.4 cm compared to the mean growth of humans by ~5 cm between the year 1900 and 2002. Swimming performance has increased in proportion to body height (Charles and Bejan 2009). Thus, the taller the athletes, the faster they can swim. Apart from anthropometry, the developing physiological factors in adolescence also seemed to have a main impact on peak swimming performance (Tanaka and Seals 2003; Zamparo 2006), such as the propelling efficiency, changes in muscle strength, biomechanical factors and bio-energetic values (Zamparo 2006; Jürimäe et al. 2007). Schulz and Curnow (1988) commented on the change in swimming performance as ‘dramatically’ within the time period of 1896 to 1980 and considered the progress of swimming performance to be a result of external factors such as ‘improved training programs and diets, better equipment, larger population bases from which athletes are selected, as well as more efficient selection strategies’ - which we can only agree with.

### Strengths and limitations of this study and implications for future research

A strength of the present study is that we included all distances and all strokes of freestyle, breaststroke, backstroke, butterfly and individual medley out of one data set in contrast to other studies investigating only single strokes. The present study is, however, limited in terms of missing data for anthropometric (Siders et al. 1993; Jagomägi and Jürimäe 2005; Zampagni et al. 2008; Charles and Bejan 2009; Lätt et al. 2010), biological (Schulz and Curnow 1988; Larsson et al. 1979; Rodrigues et al. 2006; Lata and Walia 2007), and physiological (Tanaka and Seals 2003; Zamparo 2006; Jürimäe et al. 2007) characteristics. Swimming performance and probably also the changes in the age of peak swimming performance are affected by anthropometric characteristics such as body height and body mass (Charles and Bejan 2009). Thus, the comparison between data of the aforementioned investigations in different time periods with the main findings of the present studies might also have been affected by that fact. The age of finalists at the specific events was calculated for each swimmer by the swimmer’s year of birth and the exact date of event. The exact year of birth could not have been detected for each swimmer. Thus, we assumed that the deviations from the very exact age will compensate one another by a normal distribution. Therefore, we need further research about the age of peak swimming performance, its changes over time, its differences between the sexes, and its behaviour in different strokes and distances in recent years and also at international level. Future studies should approve our findings in other competitions at world-class level such as for example the Commonwealth Games and the Pan Pacific Championships. Furthermore, we probably have not examined a time period that is large enough to assess progression in swimming performance with validity. Therefore, further research has to be done with an appropriate timeframe. Future studies that can establish a model for swimming performance with sufficient data before 2009 and after 2010 (when the FINA rules changed for the wetsuit) would be really interesting.

### Practical applications

With the knowledge of the present study athletes and coaches might be able to estimate the age of future World and Olympic Champions. Generally, there seemed to be a trend for an increase in the age of the finalists with exceptions for longer distances (200 m to 800 m) where for a few events/races a trend for a decrease in the age of the finalists occurred. However, a model for predicting the age of future champions should be established. This insight could probably give higher motivation to athletes who will be old (and young) enough when competing in important competitions to reach the finals. Therefore, the knowledge about the changes in the age of finalists over time could be ‘of large interest to the athletes and their supportive agents’ (Issurin et al. 2008). For supportive agents, new selection strategies should be done based on the age of swimmers.

## Conclusions

In summary, in the last ~20 years the age of the finalists increased in both the World Championships and the Olympic Games with some minor exceptions (200 m backstroke in women, 400 m freestyle and 200 m breaststroke in men in World Championships and 800 m freestyle in women and 400 m individual medley in men in Olympic Games) and performance of the finalists improved. Thus ‘older’ swimmers can achieve the finals in World Championships and Olympic Games and they can improve their performance.
